# Supplementary material for: Radionuclides for Targeted Therapy: Physical Properties
Source: Molecules. 2022 Aug 25;27(17):5429. doi: 10.3390/molecules27175429 (PMC9457625; doi:10.3390/molecules27175429)
Supplement: Supplementary file 1 [file molecules-27-05429-s001.zip › molecules-1820809-supplementary.pdf]

Supplementary file S1

PubMed search

A search in PubMed (https://pubmed.ncbi.nlm.nih.gov/) with the PubMed-API-module from the library pymed (https://github.com/gijswobben/pymed) was conducted. First, the 1252 radionuclides tabulated in ICRP 107 was loaded and searched with the following string:
(("radionuclide"[tiab] OR "radiopharmaceuticals"[MeSH Terms] OR "Radioisotopes/therapeutic use"[MAJR] OR "radiopharmaceutical" OR "radiopharmaceuticals" OR "radioimmunotherapy"[MeSH Terms] OR "immunoradiotherapy" OR "radioconjugate" OR "radioimmunotherapy" OR "immunoradiotherapy" OR "ELEMENT Radioisotopes/therapeutic use"[MeSH Terms] OR radioELEMENT OR "radioconjugate" OR "radiopeptide") AND ((clinical[Title/Abstract] AND trial[Title/Abstract]) OR clinical trials as topic[MeSH Terms] OR clinical trial[Publication Type] OR random*[Title/Abstract] OR random allocation[MeSH Terms] OR therapeutic use[MeSH Subheading] OR Human[MeSH Term]) NOT ("brachytherapy" OR "external beam radiotherapy" OR "heavy ion radiotherapy" OR "radioisotope teletherapy" OR "proton therapy")) AND (ElXX OR XXel OR el-XX OR XX-el OR XXELEMENT OR ELEMENT-XX OR XX-ELEMENT OR ELEMENTXX OR EL-XX)

Where ELEMT and EL-XX is the name, symbol and number of the radionuclide. The number of hits in PubMed was retrieved. The following radionuclides were removed due to primarily having diagnostic properties:

99m-Tc, 111-In, 125-I, 64-Cu, 67-Ga, 68-Ga, 18-F

The rest of the radionuclides was checked and the radionuclide removed from the data set if either:

- Only used as a target for therapeutic production
- Diagnostic tracer
- Used as a companion
- Hit due to environmental radiation/non-therapeutic
- When used as an ex-vivo generator
- Bi-product of production
- Included because of the abbreviation being interpreted as part of text
- Other non-therapeutic use

Radionuclides were kept if they had either

- Studied in a clinical/pre-clinical study
- Considered for therapeutic uses, including simulation studies
- Included in a review of radionuclide for therapeutic use

Clinical trials

ClinicalTrials.gov (https://clinicaltrials.gov/ct2/home) was used to find clinical trials involving radionuclides. The search was restricted to Interventional Studies and cancerous diseases. The previously acquired list of radionuclides with hits in PubMed in addition to 125-I, 67-Cu, and 111-In were used. Each radionuclide was searched twice, first by XX-El and then by El-XX. The results were screened and studies including radionuclides as unsealed sources were kept. Either the actual (when available) or estimated start date was retrieved, and the year recorded.

Supplementary file S2

To illustrate the effects of range on energy deposition changing with geometry and size, Monte Carlo simulations were performed with Geant4 Application for Tomographic Emission (GATE) [97,98]. Matlab R2017a was used to analyse results and to create source files. Four different sources were used: a pure alpha source with alpha energy 5.5 MeV and three pure beta sources with energies set according for the beta emission energy spectra for lutetium-177, iodine-131, and yttrium-90. The beta emission energy spectra was taken from RADAR [99] and the nuclides were chosen for the illustration as they are three most used nuclides in targeted therapy in addition to being examples of low, medium, and high energy beta emitters. The physics list used was electromagnetic standard option 4. Two different types of simulations were performed, to simulate sphere and shell sources. For all geometries, the material in every voxel was water. Spheres diameters ranged from 0.02 mm to 100 mm. For the shells, two outer diameters were used, 10 mm and 30 mm, and the thickness was varied. The dose actor and dose by regions output was used to extract energy deposited in the sphere, inside the core of the shell, in the shell, and outside.
GATE was also used to calculate X_{90} of the 15 beta-emitters most commonly published on. Beta spectra from RADAR [99] were used for all beta-emitters except erbium-169, which was taken from ICRP 107 [4] as it was not in the RADAR publication. Pure beta emitting point sources were placed in a box of water. A dose actor was used to register energy deposition. 3D Slicer (www.slicer.org) was used to find the sphere radius that enclosed 90% of the energy deposited.

References:

97. Jan S, Santin G, Strul D, Staelens S, Assié K, Autret D, et al. GATE: a simulation toolkit for PET and SPECT. Phys Med Biol. 2004;49(19):4543-61.
98. Sarrut D, Bardiès M, Boussion N, Freud N, Jan S, Létang JM, et al. A review of the use and potential of the GATE Monte Carlo simulation code for radiation therapy and dosimetry applications. Med Phys. 2014;41(6):064301.
99. RADAR – the RAdiation Dose Assessment Resource. https://www.doseinfo-radar.com/
4. Eckerman, K.; Endo, A., ICRP Publication 107. Nuclear decay data for dosimetric calculations. *Ann ICRP* **2008,** 38, (3), 7-96.
